# Clinical use, challenges, and barriers to implementation of deformable image registration in radiotherapy – the need for guidance and QA tools

**DOI:** 10.1259/bjr.20210001

**Published:** 2021-04-29

**Authors:** Mohammad Hussein, Adeyemi Akintonde, Jamie McClelland, Richard Speight, Catharine H Clark

**Affiliations:** 1 Metrology for Medical Physics Centre, National Physical Laboratory, Teddington, UK; 2 Centre for Medical Image Computing, University College London, London, UK; 3 Leeds Cancer Centre, Leeds Teaching Hospitals NHS Trust, Leeds, UK; 4 Department of Medical Physics and Biomedical Engineering, University College London Hospitals NHS Foundation Trust, London, UK; 5 Medical Physics and Biomedical Engineering, University College London, London, UK

## Abstract

**Objective:**

The aim of this study was to evaluate the current status of the clinical use of deformable image registration (DIR) in radiotherapy and to gain an understanding of the challenges faced by centres in clinical implementation of DIR, including commissioning and quality assurance (QA), and to determine the barriers faced. The goal was to inform whether additional guidance and QA tools were needed.

**Methods:**

A survey focussed on clinical use, metrics used, how centres would like to use DIR in the future and challenges faced, was designed and sent to 71 radiotherapy centres in the UK. Data were gathered specifically on which centres we using DIR clinically, which applications were being used, what commissioning and QA tests were performed, and what barriers were preventing the integration of DIR into the clinical workflow. Centres that did not use DIR clinically were encouraged to fill in the survey and were asked if they have any future plans and in what timescale.

**Results:**

51 out of 71 (70%) radiotherapy centres responded. 47 centres reported access to a commercial software that could perform DIR. 20 centres already used DIR clinically, and 22 centres had plans to implement an application of DIR within 3 years of the survey. The most common clinical application of DIR was to propagate contours from one scan to another (19 centres). In each of the applications, the types of commissioning and QA tests performed varied depending on the type of application and between centres. Some of the key barriers were determining when a DIR was satisfactory including which metrics to use, and lack of resources.

**Conclusion:**

The survey results highlighted that there is a need for additional guidelines, training, better tools for commissioning DIR software and for the QA of registration results, which should include developing or recommending which quantitative metrics to use.

**Advances in knowledge:**

This survey has given a useful picture of the clinical use and lack of use of DIR in UK radiotherapy centres. The survey provided useful insight into how centres commission and QA DIR applications, especially the variability among centres. It was also possible to highlight key barriers to implementation and determine factors that may help overcome this which include the need for additional guidance specific to different applications, better tools and metrics.

## Introduction

Deformable image registration (DIR) is the process of non-rigidly aligning one image to another, into a common spatial coordinate frame, in order to account for anatomical differences between the two images.^1^ Recently, the use of DIR has become more common in radiotherapy, in particular for adaptive radiotherapy,^2,3^ where treatment is adapted to account for anatomical changes in between treatment fractions. In some adaptive radiotherapy workflows, DIR is becoming an important component and, as a result, DIR algorithms are becoming increasingly available in commercial treatment planning systems.

Various applications of DIR in radiotherapy have been proposed. Multimodal pre-treatment images are often used during planning to combine information from different imaging modality to aid in the delineation of tumour volumes or organs at risks (OARs).^4,5^ Another application is contour propagation, here the planning contours are propagated to repeat CT images acquired during treatment, thereby saving delineation time.^6–8^ Treatment adaptation methods such as the “dose of the day” are another application, which are based on creating a deformed CT from the planning CT and cone beam CT (CBCT). The deformed CT maps the Hounsfield unit (HU) information from the planning CT while the anatomical information is obtained from the CBCT.^9–12^ This can be used to estimate the dose delivered on the day of treatment. Finally, dose accumulation is another potential application where DIR could be used, here quantification of the dose absorbed over the course of treatment fractions is estimated by warping the dose to a common reference anatomy.^13,14^ This approach could be used to estimate the discrepancies between the planned and the delivered dose.

The results obtained from a DIR algorithm can contain uncertainties, therefore care should be taken when using the deformation obtained to represent anatomical changes accurately. The registration can be affected by several factors such as the type of imaging modality used, homogeneity of regions, changes in tissue, artefacts in images, and the type of DIR algorithm used.^3^ Therefore, it is critical that DIR algorithms are validated carefully before they can be used clinically.

Multiple studies have been conducted to validate the reliability of different DIR algorithms. Veiga et al^9^, compared the performance of different DIR algorithms for dose warping with H&N patients, and they found that the choice of DIR algorithm leads to uncertainties in dose warping. Nie et al^15^ performed a similar study using three different commercial DIR methods on H&N, prostate and cranial spinal irradiation (CSI). They also concluded that there were variability between the different DIR algorithms used. Other comparative studies have also demonstrated that there can be discrepancies between different DIR algorithm.^16,17^

Multi-institutional studies have also been performed to validate the use of DIR algorithms. Kadoya et al^18^ evaluated commercially available DIR software using 4DCT thoracic images from multiple centres, they found that there can be variation in the DIR performance among different institutions as a result of differences in procedure and the DIR algorithm used. Loi et al^19^ also performed a similar study which included 13 different institutions, and they found that the accuracy of the algorithm was site-specific.

The results from these different studies highlighted the need for a robust assessment of the DIR software for each clinical scenario and institution. As a result, a recent publication by the AAPM TG 132^3^ aimed to tackle this issue. This publication provided quality assurance guidelines for the use of DIR in radiotherapy, which highlighted the need to quantify the quality of the registration in order to assess if it is applicable in a clinical scenario. The report also detailed the need for formal quality management of the uncertainties of DIR. However, the report does not provide specific guidelines for advanced DIR applications such as dose deformation.

A current significant challenge for radiotherapy departments is in the effective implementation of, and confidence in assuring use of DIR algorithms.^3^ Yuen et al^20^ published a survey of 57 international centres to identify the variation, implementation, and decision-making criteria for the clinical use of rigid image registration and DIR in 2018. A known limitation of the survey was the limited response in Europe, which may have led to under- or overestimation of clinical adoption in this region. Kadoya et al^21^ performed a survey in 161 centres in Japan looking at the clinical use of different DIR applications and commissioning methods. While these studies provided an insight into the clinical use of image registration, further detailed information was needed on the type of ongoing QA..

Therefore, the aim of this study was to conduct a survey focussed on metrics used, how centres would like to use DIR in the future and challenges faced, in order to assess the current status of the use of DIR software. Data were gathered from UK centres, specifically on which centres were using DIR clinically, which applications were being used, what commissioning and QA tests were performed, and what barriers were preventing the integration of DIR into the clinical workflow. An additional aim of this survey was to determine whether additional guidance and QA tools were needed.

## Methods and materials

### Definition of DIR and applications in radiotherapy

At the outset, DIR software was defined as: “software that explicitly performs a deformable registration and saves the resulting transformation and/or deformed images to be further used for some other application”. The following applications of DIR in radiotherapy were defined: (i) propagation of contours from one scan to another (*e.g.* rescan or atlas-based autocontouring).

(ii) Fusing/combining pre-treatment multimodality images (*e.g.* target delineation with PET or MRI).
(iii) Propagation of dose (such as for dose accumulation, previous treatments etc.) (iv) Deformation of the planning CT to daily imaging (*e.g.* daily CBCT, MVCT, MRI) to evaluate dose coverage, to inform plan adaptation etc.

### Structure of the survey

**Figure 1.**
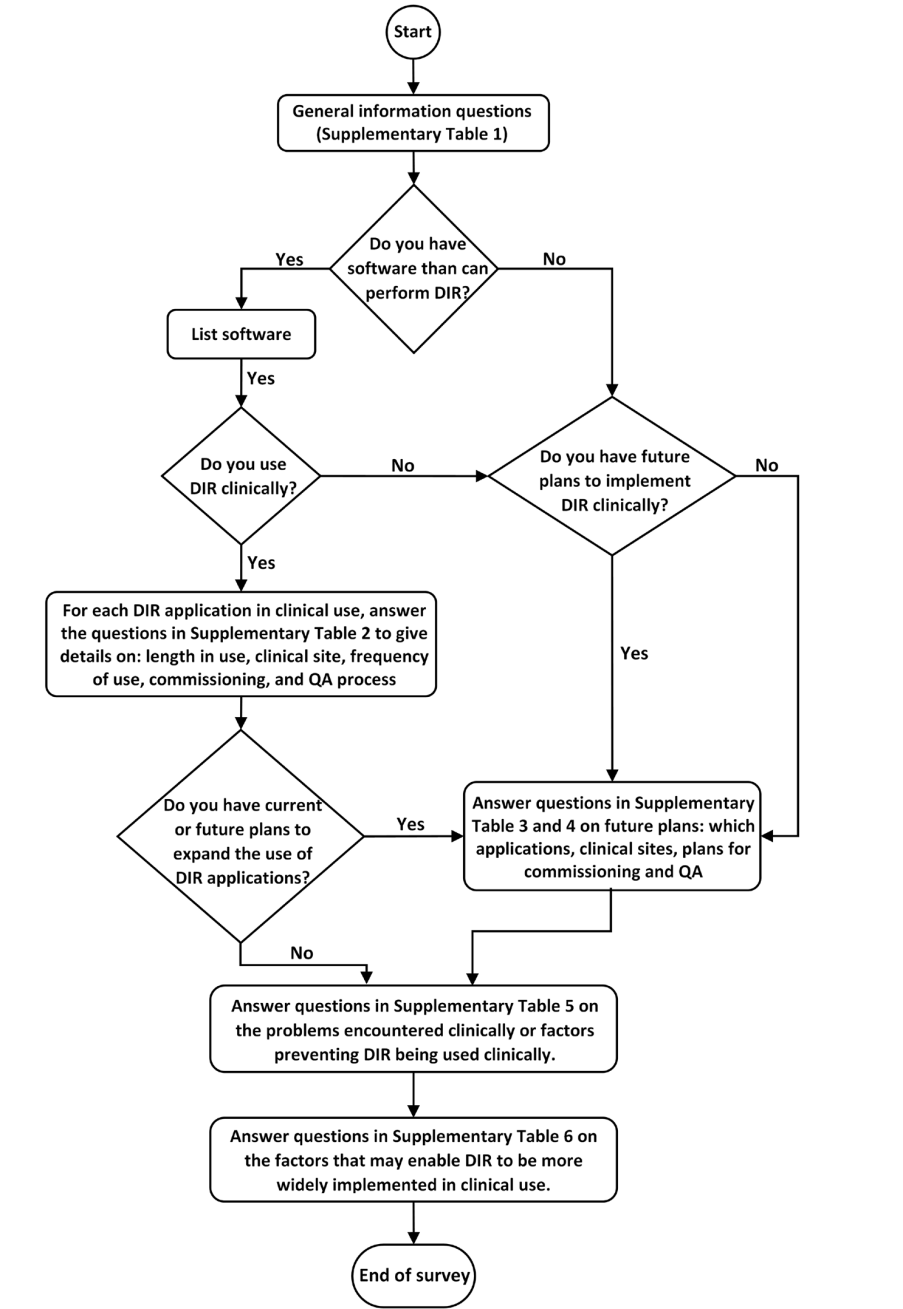
Schematic flowchart of the survey. DIR, deformable imageregistration

The survey was constructed in SurveyMonkey^™^ with logic features used to take users to different parts of the survey depending on their answers. Figure 1 shows a flow chart of the survey. Broadly, the survey was setup along six themes. The first theme of the survey included general initial sections to fill in basic demographics and whether software for performing DIR was available in the hospital. The second section was whether centres have software that can perform DIR, whether DIR was used clinically, which clinical applications DIR algorithms were used for, and which anatomical sites they were used for. The third section was which type of commissioning tests and data were used, and what routine QA practice and frequency was being performed. The fourth theme was which clinical applications UK centres would like to use DIR for in the future. The fifth theme was to understand what the challenges and barriers to the use of DIR in clinical practice were. The final theme focussed on which factors could enable centres to use more DIR in clinical practice. The full survey questions are given in Supplementary Tables 1–6.

### Survey dissemination

The link to the survey and a covering letter were emailed to the Heads of Radiotherapy Physics of 71 radiotherapy service providers in the UK (68 public and 3 were private). Providers that had satellite centres were counted as a single site. The covering letter made it clear that centres that did not use DIR clinically were encouraged to fill in survey. The survey was opened for data collection between January and April 2019. Email reminders were sent to all Heads of Radiotherapy Physics twice during this time.

### Specific components of the survey

#### Theme 1: general initial questions

The number of linacs was used as a surrogate for department size. Centres filled in their name and contact details. All surveyed centres were asked if they have any software that can perform DIR and which software (manufacturer and type), with the definition of DIR included as part of this question. All centres were subsequently asked: “Do you currently use DIR clinically?”.

#### Theme 2: which DIR applications are being used clinically?

Centres using DIR clinically were asked if they use each of the four DIR applications (For each application, the questions and answer options are listed in Supplementary Table 2). In summary, the questions were posed to gather information on how long the application has been used for, frequency of clinical use, and for which clinical sites.

For the registration of pre-treatment with multimodality imaging applications, users were additionally asked to list which image modalities are registered. For the dose propagation application, centres were asked the purpose for which it is used: for previous treatment dose overlap calculation, to perform dose accumulation to inform adaptive RT and/or replanning or to perform dose accumulation for estimating delivered dose when conducting dose–outcome studies.

The survey gave the option to add up to three DIR applications that we did not explicitly specify in the prescribed questions. For any optional application, the responder was asked to summarise how long they have used it, how often, which clinical sites, what commissioning tests they did, and what ongoing QA was being performed.

Subsequently, centres were asked to identify the challenges that they faced in the clinical implementation of DIR (Supplementary Table 3).

#### Theme 3: what type of commissioning and QA is performed?

Centres using DIR clinically were asked what type of commissioning was done and what ongoing QA was being performed, including which type of data are used and what tests/metrics are measured (see Supplementary Table 2). For commissioning and QA, the centres were requested to identify which guidelines or recommendations were followed.

The commissioning and QA tests given as options are shown Table 1.^1,3^

**Table 1.** Types of commissioning and QA tests

Type	Description
A	Qualitative assessment of registered image, this typical involve using visualization technique such as examining the difference image between the registered image and the reference image.
B	Qualitative assessment of DVF.
C	Qualitative assessment of contours on registered images such as overlaying anatomical structures defined on one image, and these can be overlaid on another image.
D	Quantitative assessment of contours on registered images using metrics such as DICE coefficient and Hausdorff distance.
E	Quantitative assessment of landmark alignment using TRE.
F	Assessment of Jacobian determinant
G	Assessment of other DVF metrics.
H	Consistency and transitivity measurements, these techniques include performing the registration in both directions to ensure that the registration is inverse consistent.
I	Quantitative assessment using digital phantoms, these can also be useful to quantitatively assess the accuracy of image registration.
J	End to end tests using physical phantoms, this ensures that all the different part of the radiotherapy system workflow works accurately.

DVF, deformation vector field; QA, quality assurance; TRE, target registration error.

#### Theme 4: which DIR applications would the centres like to use clinically in the future?

Centres who were already clinical were asked if they had any plans to expand their clinical use of DIR. For centres that were not using DIR clinically at the time of the survey, they were asked if they had intentions to implement it clinically in the future. All centres that answered ‘Yes’ to these questions were asked the questions in Supplementary Tables 3 and 4, if their timescale was within 1 year, 1–3 years, >3 years. Centres were asked which anatomical sites they planned to implement DIR for, and if they had any plans in place for commissioning and/or ongoing QA.

#### Theme 5: what are the perceived challenges and barriers to the clinical use of DIR?

All centres were asked to identify what challenges they have faced in implementing DIR clinically or what are the perceived barriers preventing or hindering implementation. The full list of options is given in Supplementary Table 5. Options for selection included: determining when a registration is satisfactory, determining what to do when registration is not satisfactory, determining qualitative methods of ensuring deformation is accurate, lack of knowledge locally, lack of guidance document etc. There was also a free text box for centres to add their own additional comments.

#### Theme 6: which factors could enable centres to use DIR more in clinical practice?

The final section of the survey sought to find out which factors may help facilitate or give more confidence in implementing DIR into clinical practice. The full list of options is given in Supplementary Table 6. Such interventions may include clear guidelines on how to use registration for different applications, training courses, better tools or metrics for commissioning registration software, better tools or metrics for QA of registration results. All centres were asked to fill in this section of the survey.

## Results

### Theme 1: general responses

**Figure 2.**
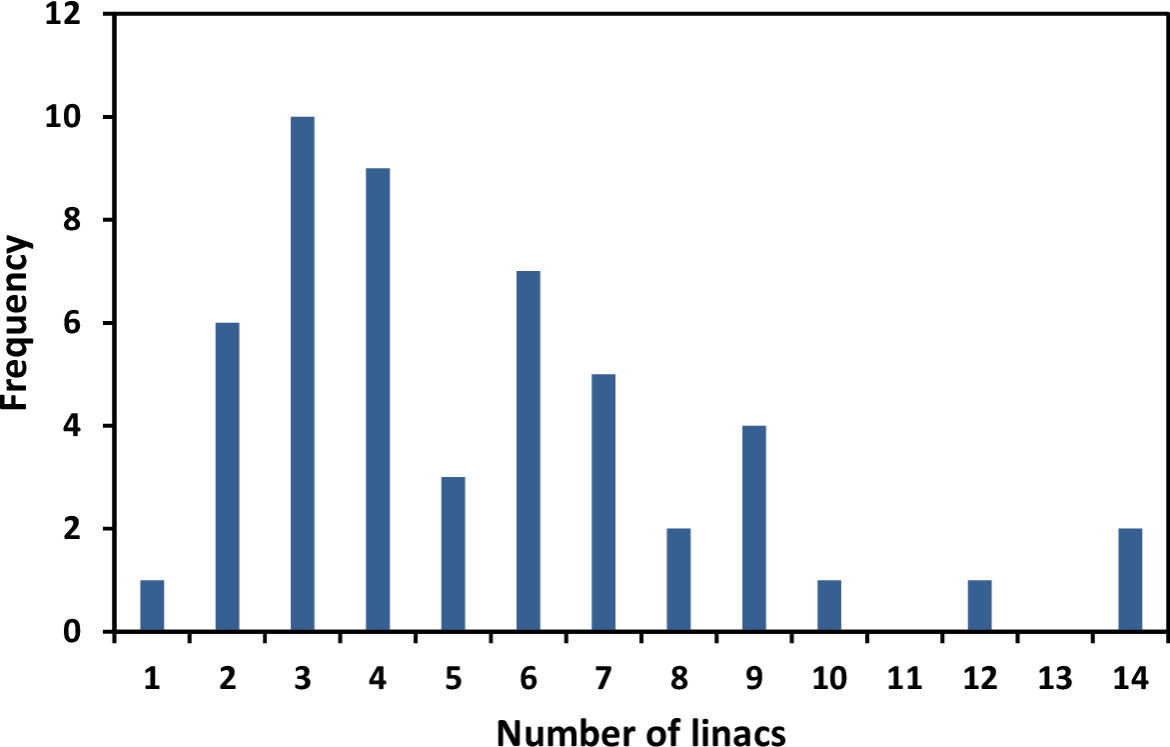
Histogram of number of linacs in responding centres.

**Figure 3.**
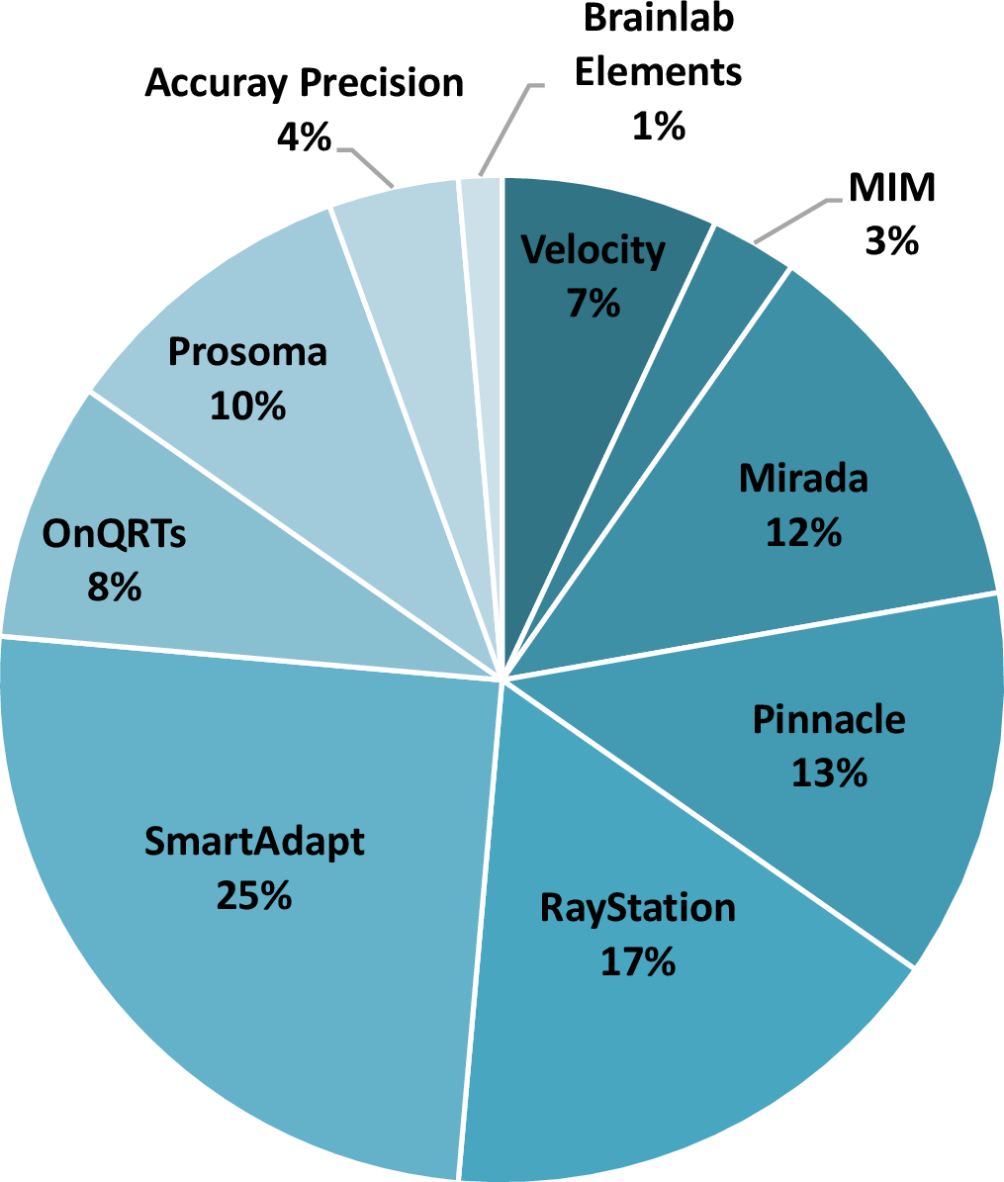
DIR capable software in use. The percentages are relative to a total of 72 software installed in responding centres. DIR, deformable imageregistration

In total, 51 (73%) of surveyed UK centres responded, 49 were public centres and 2 were private. Figure 2 shows the histogram of the number of linacs in these centres which was used as a surrogate of department size, showing a spread in small to large centres that responded to the survey. From the responding centres, 47 of 51 (92%) reported access to at least one commercial software that could perform DIR according to our definition. Some centres had access to more than one software leading to a total of 72 software installed in responding centres. Figure 3 shows the distribution of software in the centres, with Varian SmartAdapt the most commonly installed. In total, 20 of 51 (39%) centres indicated that they already used DIR clinically. From the remaining 31 of 51 centres, 23 (45%) centres had plans to implement an application of DIR within 1 year of responding to the survey.

### Theme 2: DIR applications being used clinically

Table 2 shows a summary of the different applications of DIR in clinical use, including the most common clinical sites, type of commissioning data used and frequency of ongoing QA.

**Table 2.** Summary of the different applications that were used clinically, including the most common clinical sites, type of commissioning data used and frequency of ongoing QA

Application	Number of centres (% of 20 clinical users)	Most common anatomical sites	Length of time in clinical use	Types of commissioning data	Ongoing QA frequency
Propagate contours from one scan to another	19 (95%)	Head & neck (17) Prostate (12) Lung, rectum, gynae (eight each)	<1 year (4) 1–3 years (11) >3 years (4)	Patient (13) Digital phantom (4) Physical phantom (3)	Patient-specific (13) None (6)
Registering pre-treatment multimodality imaging (*e.g.,* target delineation with PET or MRI)	10 (50%)	Head & neck (7) Brain, liver, prostate, gynae (two each)	<1 year (3) 1–3 years (4) >3 years (3)	Patient (7) Digital phantom (3) Physical phantom (1)	Patient-specific (6) Monthly (1) Annual (1) None (3)
Deform planning CT to daily images	8 (40%)	Head & Neck (6) Lung, liver, prostate, Gynae (four each)	<1 year (1) 1–3 years (7) >3 years (0)	Patient (7) Digital phantom (2) Physical phantom (1)	Patient-specific (6) None (2)
Dose propagation	7 (35%)	Head & Neck (6), lung (4), prostate (4)	<1 year (1) 1–3 years (6) >3 years (0)	Patient (5) Digital phantom (2) Physical phantom (1)	Patient-specific (6) None (1)

PET, positron emission tomography; QA, quality assurance.

The most common application of DIR in clinical use was to propagate contours from one scan to another, with 19 out of 20 centres using it. Across all the applications, the most common anatomical site was head & neck. Centres experience also varied depending on the application with longer experience (>3 years) reported for contour propagation and pre-treatment multimodality imaging DIR, whereas for dose propagation and deforming the planning CT to daily images no centre reported experience was 3 years or more.

All but one of the 10 centres using DIR to register pre-treatment multimodality imaging used it for MR to CT registration. Four centres used it for PET-CT to planning CT registration and three used it for diagnostic CT to planning CT registration.

For dose propagation, all seven centres used it for previous treatment overlap dose calculation. Five of the seven centres used it to perform dose accumulation to inform adaptive radiotherapy and/or replanning.

Seven out of eight centres used DIR to deform the planning CT to daily linac on-board CBCT to evaluate dose coverage, to inform plan adaptation etc. The eighth centre used it to deform planning CT to daily Tomotherapy MV CT.

### Theme 3: type of commissioning and QA performed

**Figure 4.**
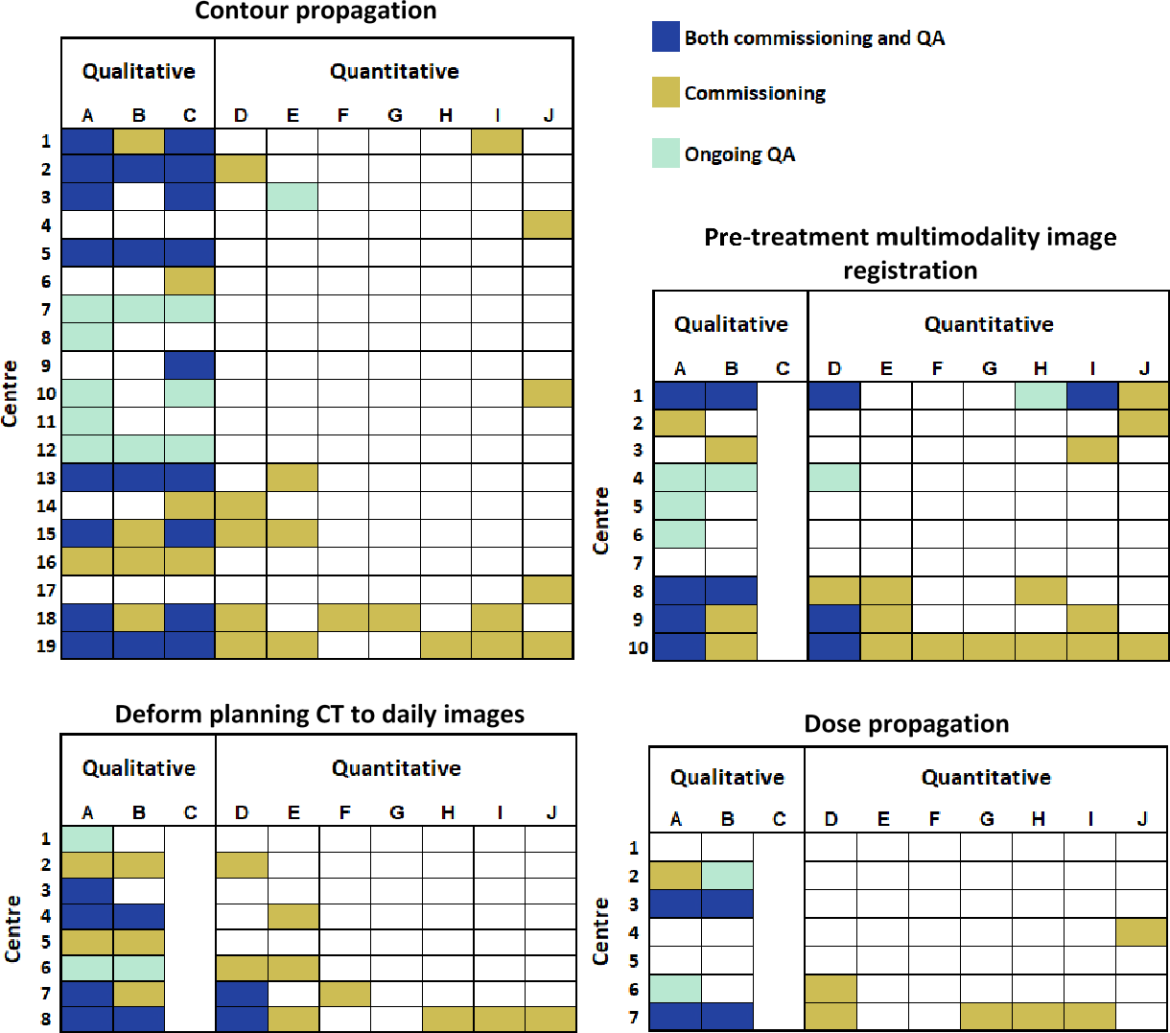
Centre-by-centre breakdown of which tests where used for each of the DIR applications. The tests A–J were defined in Table 1 and the figures are split to distinguish qualitative and quantitative tests. The figures show which centres used each respective test for both commissioning and ongoing QA, or commissioning only, or ongoing QA only, or none (empty boxes). DIR, deformable image registration; QA, quality assurance.

Table 2 shows that in all DIR applications patient data were the most frequently used for commissioning tests. Patient-specific QA was most frequently used for ongoing QA. The types of tests performed varied depending on the type of application, with qualitative tests being more common for ongoing QA. Figure 4 shows centre-by-centre breakdown of which tests were used for both commissioning and ongoing QA, commissioning only, or QA only. The different tests were defined in the methodology, however in summary, tests A–C were qualitative tests, and D–J were quantitative. In all applications, there is an apparent tendency to use qualitative tests more frequently. There is some evidence, seen both in the frequency plot and the centre-by-centre breakdown, of a wider range of tests being used more frequently for commissioning. This was clearer in the DIR for contour propagation application which had the most responding centres, whereas ongoing QA was mainly qualitative.

The centre-by-centre data breakdown is shown in Figure 4. 21% (4/19) of centres used DIR for contour propagation, 40% (4/10) for multimodality pre-treatment image registration, 12.5% (1/8) for registering the planning CT to daily imaging, and 28.5% (2/7) for dose propagation but performed ongoing QA rather than commissioning (with the exception of three centres that performed neither). Conversely 26% (5/19) of centres used DIR for contour propagation, 20% (2/10) for multimodality pre-treatment image registration, 25% (2/8) for registering the planning CT to daily imaging, and 14% (1/7) for dose propagation and performed commissioning tests only.

The guidelines used by centres were AAPM TG132 (8 out of 20 centres), published journal articles (8 out of 20 centres). One centre used manufacturer recommendations. 5 out of 20 centres indicated none was used.

### Theme 4: which DIR applications would centres like to use clinically in the future?

**Figure 5.**
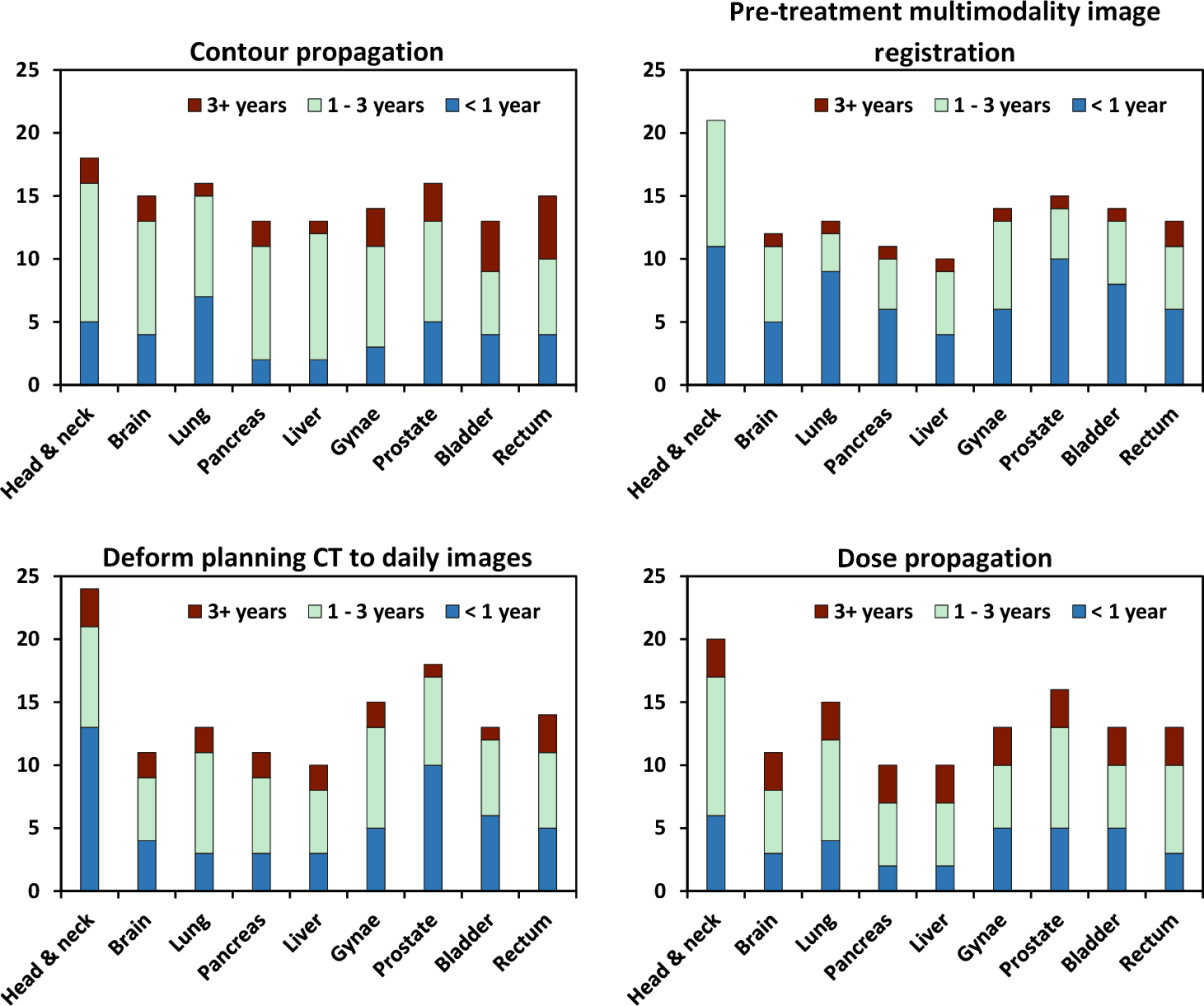
Future plans by responding centres for each DIR application per anatomical site. The y-axis is the number of responding centres. DIR, deformable imageregistration.

A total of 38 out of 51 responding centres (75%) had plans to either expand their use of DIR application or implement DIR within 1 year post survey (15 centres that were already clinical, and 23 centres who were not already clinical at time of responding). The number of centres per DIR application were as follows: 33 of 38 (87%) centres planned to use DIR for contour propagation from one scan to another, 26 of 38 (68%) centres for registering pre-treatment multimodality imaging, 29 of 38 (76%) centres for dose propagation, and 30 of 38 (79%) centres for deforming the planning CT to daily images. Within the latter, 29 out of 30 centres planned to deform the planning CT to daily linac on-board CBCT, one centre to MRI, and 1 centre to repeated planning CTs. Figure 5 gives details on which anatomical sites centres are planning to use each DIR application for and plotted based on anticipated timescale for implementation; <1 year, 1–3 years, and >3 years post survey.

**Figure 6.**
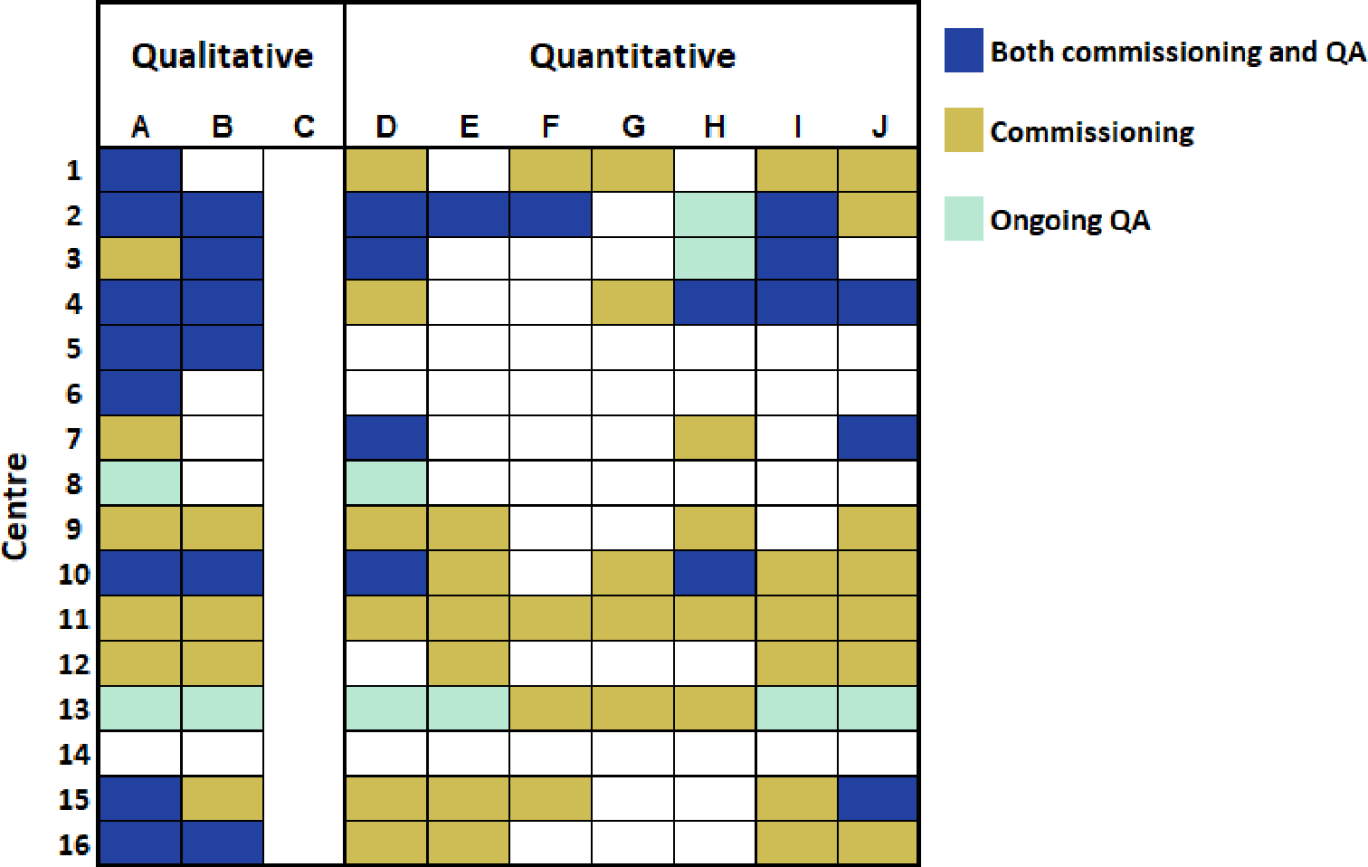
Type of commissioning & validation, and ongoing QA tests that centres are planning to perform when expanding or clinically implementing DIR, shown as a centre-by-centre breakdown which shows which centres planned to use each particular test A-J (see Table 1) for both commissioning and ongoing QA, commissioning only, and ongoing QA only. DIR, deformable imageregistration; QA, quality assurance.

16 out of 38 (42%) of centres had plans for how to commission and validate their future applications. 15 centres planned to use patient data, 12 planned to use digital phantoms and 9 planned to use physical phantoms. Figure 6 shows the type of commissioning & validation, and ongoing QA tests that centres are planning to perform both as a frequency plot and on a centre-by-centre breakdown. Similar to the results that were shown in Figure 4, there is a trend to planned use of a wider range of tests for commissioning. 1 out of the 16 centres indicated that they had plans but commented that it was too early for this to give specific details of which tests they would use. One centre chose all of the tests and commented that they planned to carry out all of the tests recommended in TG132.

### Theme 5: challenges and barriers to the implementation and clinical use of DIR

Table 3 shows a summary of the common challenges and factors that present barriers in the clinical implementation and use of DIR. This includes answers from all 51 centres that responded to the survey. The most common challenge was lack of staff resources (63% of responders), however, a large variety in challenges was faced at different centres, with most challenges encountered at multiple centres, and only one centre responding that they faced no challenges.

**Table 3.** Summary of the key challenges that cause barriers in clinical adoption and use of DIR

Answer Choices	Responses	
Lack of time or staff resource	63%	32
Determining quantitative methods of ensuring deformation is OK	51%	26
Lack of deformable physical phantoms	51%	26
Determining when a registration is satisfactory	45%	23
Lack of guidance document	41%	21
Determining what to do when registration is not satisfactory	39%	20
Determining qualitative methods of ensuring deformation is OK	39%	20
Lack of knowledge locally	33%	17
Software DIR results not acceptable	29%	15
Lack of software, or funds for software	26%	13
Lack of tariff for using DIR in the clinic	24%	12
Lack of local interest from clinicians	18%	9
Software user friendliness	4%	2
Use cases not always clear	2%	1
Lack of training and/or workshops	2%	1
None	2%	1

DIR, deformable image registration.

The percentages are relative to the 51 centres who responded to the survey.

Specific challenges from the 20 respondents who were using DIR clinically at the time of the survey are given in Table 4. These included the need for improved user friendliness of QA software, difficulty implementing existing QA recommendations and determining when a deformable registration was satisfactory.

**Table 4.** Specific challenges faced by centres in the clinical implementation of DIR

Answer choices	Responses	
Lack of appropriate tools for commissioning	55%	11
Lack of guidance at the time	50%	10
Software capability	40%	8
Did not know what tolerances to accept	25%	5
None	10%	2
No manufacturer recommendations	5%	1

DIR, deformable image registration.

The percentages are relative to the 20 centres who were using DIR clinically at the time of the survey.

### Theme 6: factors which could enable centres to use DIR more in clinical practice

Table 5 gives a summary of the factors that may allow centres to use DIR more in clinical practice. This shows the most common responses (67%) related to clear guidelines on how to use DIR for different applications, better tools for commissioning and QA. Other responses included the need to improve DIR algorithms, need for improved user friendliness of QA software, difficulty implementing existing QA recommendations, and more detailed studies benchmarking software with written information on how they work, strengths and weakness.

**Table 5.** Summary of the factors that may allow centres to use DIR more in clinical practice

Answer choices	Responses	
Clear guidelines on how to use registration for different applications	67%	34
Better tools for commissioning registration software	67%	34
Better tools for QA of registration results	67%	34
Training courses	55%	28
Better registration software and smoother workflows	6%	3
Improved accuracy of DIR algorithms	4%	2
Ability to quantify uncertainty	2%	1
External funding	2%	1
Evidence of efficacy	2%	1
Written info on how the software works, its strengths, weaknesses and potential pitfalls	2%	1
None	2%	1

DIR, deformable image registration; QA, quality assurance.

The percentages are relative to the 51 centres who responded to the survey.

## Discussion

The results of the survey indicated that, whilst almost all the responding UK centres had access to software capable of DIR, around two-thirds were not using it in routine clinical practice. Most clinical experience was within 3 years from the time of the survey. The most common DIR application in clinical use was contour propagation (95%) whereas DIR for dose propagation was the least used (35%). This contrasts with the surveys of Yuen et al^20^ and Kadoya et al^21^ who reported that contour propagation was used by 47 and 53% of centres respectively, and dose propagation was used by 63 and 73% respectively. It was not possible to directly compare DIR for multimodality pre-treatment image registration and DIR for deforming daily imaging as the surveys by Yuen et al^20^ and Kadoya et al^21^ did not explicitly separate them. Head & neck was the most popular anatomical site for centres using DIR clinically and for future implementation. This was also the case in other surveys. This is likely due to additional focus on adaptive radiotherapy and where this technique has already been implemented manually, *e.g.* replanning for weight loss or neck mass reduction. The data show that a future application that appears to be gaining further attention is in the UK dose propagation. The latter is an area of controversy^22,23^ and will need suitable recommendations that can guide centres. A key challenge with dose propagation (as opposed to the other applications) is the combination of uncertainties in the deformation, propagation, and calculation, and hence there is caution surrounding the use of this application.

The survey gave useful insight into how centres commission and QA DIR applications. As shown in Figure 4, commissioning and ongoing QA can vary among centres for the same DIR application. Some centres performed multiple qualitative and quantitative commissioning tests. Others performed no formal commissioning but relied on establishing ongoing QA. Some centres performed commissioning but no ongoing QA, presumably confident that these were not needed after the software was validated. Of note is that three centres did not perform either commissioning or ongoing QA. These were for the applications registering pre-treatment multimodality imaging and dose propagation. Similar surveys showed lack of standardisation of commissioning tests and qualitative tests were more common.^20,21^ These may be due to several reasons such as lack of appropriate guidance at the time of commissioning, difficulty following existing guidance for those applications, lack of access to appropriate tools or data sets. The most common data set used for commissioning was patient data, with limited use of physical phantoms. Reasons for this could be due to lack of availability of suitable deformable phantoms that are representative of DIR algorithm behaviour in patient data, lack of awareness of their existence, or are unsuitable for the imaging modalities used.

Key barriers to adoption included lack of time or staff resource, determining when a deformable registration was satisfactory, determining quantitative methods for ensuring DIR is acceptable. Almost unanimously, ongoing QA tests were qualitative and tended to be patient-specific. This could be a result of difficulty in knowing which quantitative tests and metrics are appropriate for ongoing QA, or lack of access to appropriate software or tools. Also, quantitative patient-specific QA requires a ‘ground truth’ to be known for comparison. For example, for quantitative contour assessment, tools such as dice similarity coefficient are commonly available in commercial treatment planning system that have DIR algorithms but are limited as they do not provide information about volume shape and size, and hence better measures are required which give clinically relevant interpretation of the accuracy of the registration.^24^ There were also barriers related to the software in use. 15 of 51 centres indicated they found DIR results were not acceptable, and therefore there is a need for improved DIR algorithms. Smoother workflows may aid in minimising issues with lack of staff resources. Additionally, a better understanding of how the software works including strengths and weaknesses, and potential pitfalls will help users understand the limitations of the software. This shows that more help from industry is needed to develop and implement analysis tools in clinical treatment planning systems. Interestingly, 9 out of 51 centres indicate a lack of interest from clinicians, and therefore there may be a need for a clear summary of where DIR may or may not help that is accessible for the wider radiotherapy multidisciplinary team.

In the clinic, it would be useful to have an automatic tool to assess registration accuracy in order to filter out unacceptable registrations before being used.^25^ This is non-trivial as automated analysis must be quick and performed in a situation where a ground truth is not known. Several groups have proposed techniques to automatically assess image registration quantitatively when no ground truth is known.^26–28^ As shown in Figure 6, there were more centres interested in using quantitative metrics in the future. There is also a future interest in centres using end-to-end testing using physical phantoms as part of the commissioning and validation and interestingly in using this for ongoing QA. Several physical phantoms have been proposed in the literature,^24,29,30^ however, the availability of suitable phantoms is a challenge to be addressed, and requires the support of phantom manufacturers. As adaptive radiotherapy becomes increasingly taken up in clinical trials and clinical practice, multicentre dosimetry audits incorporating the assessment of the dosimetric impact and uncertainty of the use of DIR in end-to-end tests could be one approach to building confidence. Dosimetry audits have played a crucial role in the uptake of advanced radiotherapy techniques.^30^ There was also interest in using digital phantoms, some of which are already available from TG132 and they can also be made from patient data. This indicates that there is a need for standardised approaches between different centres, and specific guidelines for different applications to supplement overarching DIR guidelines such as TG132.

Therefore, further guidance is needed to address these issues and consider what is feasible for commissioning, where it is possible to produce gold-standard data (such as contours, scans, artificial deformation vector fields), and what is feasible for ongoing QA where no such data are available. This is particularly highlighted by the variability in the types of commissioning and QA tests that different clinical centres perform. All DIR applications should have some form of formal commissioning performed to ensure the tool used is fit for purpose, and some form of ongoing QA (even a simple visual inspection, formally documented) should be performed. In particular, as lack of time or staff resources was a key barrier, these need to consider the ease of use and this strengthens the argument for guidance to be specific and focussed on each application. These were also highlighted factors which may allow wider uptake of DIR in clinical practice. Guidelines should consider cases where it may not be appropriate to have hard tolerances for QA where particularly there is clear benefit of using DIR, and where careful and safe qualitative clinical judgment is required by a suitably qualified person. Examples of this are cases with large deformations or deciding on overlap treatment. Training and evidence of competency for these scenarios will be crucial.

It is acknowledged that when the detailed survey was completed in 2019, most centres that had the intention to expand or implement DIR applications in the future planned to do so in the 3 years post-survey but a proportion intended to do so in less than a year. Therefore, a short follow-up survey between February and March 2021 was conducted to check the current status, the results of this are given in the Supplementary material (Supplementary Report). This indicated that around two-thirds of centres, that had plans to expand or implement DIR applications in 2019, did not make any changes. In the centres that were clinical in 2019, one-third expanded their DIR application. The majority have plans on hold or delayed for reasons including lack of staff or resources and impact of the Covid-19 pandemic. However, 25% of centres did expand an existing DIR application or implemented a new DIR technique. No responding centre made changes to commissioning or QA techniques. It was noted that one centre implemented a new DIR technique as a result of installation of a Varian Ethos. It should be noted that machines such as the Varian Ethos or the Elekta Unity MR-Linac are designed to drive adaptive radiotherapy workflows but are still in the early stages of clinical adoption. It is anticipated that this will increase over the years, and it is likely that the software workflows developed could be made available on conventional linacs, raising the importance and immediate need for guidance on DIR commissioning and validation.

It is also acknowledged that existing DIR algorithms may be overtaken by Artificial Intelligence approaches for certain applications,^31–33^ *e.g.* contour propagation based on deep learning. However, the result of these algorithms is still subject to similar commissioning and QA challenges and therefore additional guidance should be applicable to both existing DIR algorithms and future developments in AI. Based on the results of the survey, we have started investigating guidance to address the dose-of-the-day topic as with highlighted by a few centres as a future topic of interest and is one where there is still a limited consensus.

## Conclusions

The results of the survey highlighted that there is a need for additional guidelines, better tools for commissioning DIR software and better tools for the QA of registration results, which should include developing or recommending which quantitative metrics to use. Lack of time or staff resources were the most commonly selected barrier to clinical adoption and use of DIR. This may be as centres feel they need to spend more time on commissioning and validation due to the existing guidelines and recommendations available. Therefore, guidelines need to be written with efficiency and ease of use in mind. This also supports the need for guidelines to be application-specific.
